# Towards a Corpus (and Language)-Independent Screening of Parkinson’s Disease from Voice and Speech through Domain Adaptation

**DOI:** 10.3390/bioengineering10111316

**Published:** 2023-11-15

**Authors:** Emiro J. Ibarra, Julián D. Arias-Londoño, Matías Zañartu, Juan I. Godino-Llorente

**Affiliations:** 1 Department of Electronic Engineering, Universidad Técnica Federico Santa María, Avenida España 1680, Casilla 110-V, Valparaíso 2390123, Chile; emiro.ibarra@sansano.usm.cl (E.J.I.); matias.zanartu@usm.cl (M.Z.); 2 Escuela Técnica Superior de Ingeneiros de Telecomunicación, Universidad Politécnica de Madrid, Avda, Ciudad Universitaria, 30, 28040 Madrid, Spain; julian.arias@upm.es

**Keywords:** convolutional neural networks, deep learning, domain adversarial, Parkinson’s disease, transfer learning, corpus independence, shortcut learning

## Abstract

End-to-end deep learning models have shown promising results for the automatic screening of Parkinson’s disease by voice and speech. However, these models often suffer degradation in their performance when applied to scenarios involving multiple corpora. In addition, they also show corpus-dependent clusterings. These facts indicate a lack of generalisation or the presence of certain shortcuts in the decision, and also suggest the need for developing new corpus-independent models. In this respect, this work explores the use of domain adversarial training as a viable strategy to develop models that retain their discriminative capacity to detect Parkinson’s disease across diverse datasets. The paper presents three deep learning architectures and their domain adversarial counterparts. The models were evaluated with sustained vowels and diadochokinetic recordings extracted from four corpora with different demographics, dialects or languages, and recording conditions. The results showed that the space distribution of the embedding features extracted by the domain adversarial networks exhibits a higher intra-class cohesion. This behaviour is supported by a decrease in the variability and inter-domain divergence computed within each class. The findings suggest that domain adversarial networks are able to learn the common characteristics present in Parkinsonian voice and speech, which are supposed to be corpus, and consequently, language independent. Overall, this effort provides evidence that domain adaptation techniques refine the existing end-to-end deep learning approaches for Parkinson’s disease detection from voice and speech, achieving more generalizable models.

## 1. Introduction

Parkinson’s Disease (PD) is a neurodegenerative disorder caused by the gradual death of dopaminergic neurons in the substantia nigra [1]. The impact of this neurodegenerative condition on speech is characterised by a reduced vocal loudness, a monotonous voice, a reduced fundamental frequency range, imprecise consonants and vowels, breathiness, and inappropriate pauses [2]. These symptoms are collectively known as hypokinetic dysarthria, and typically appear early in most patients with PD [3]. As a result, speech-based and voice-based assessments of PD have become an essential research topic for providing early diagnoses of PD. The primary advantage of automatic PD screening tools lies in their ability to offer a non-invasive diagnosis, which can enable timely screening applications and facilitate remote health monitoring [4].

In recent years, extensive work has been published, suggesting the potential of voice and speech characteristics as biomarkers for the development of automatic screening tools for PD. Among the state-of-the-art contributions are those based on traditional machine learning approaches, such as support vector machines, random forests, k-nearest neighbours, regression trees, and naïve Bayes [4,5,6,7,8,9,10]. These methods were trained using both acoustic features (including certain variants of the jitter, shimmer, and harmonic-to-noise ratio) and complexity measurements to model the influence of PD on patient phonation [11]. However, recent studies that incorporate Mel-frequency cepstral coefficients have significantly improved the accuracy and specificity of classification [12].

In addition to traditional machine learning algorithms, deep learning (DL) approaches are gaining considerable popularity because of their ability to exploit high-level abstract representations from not only the voice, but also the speech. DL techniques reported for PD detection include mappings from handcrafted acoustic features to output labels (PD/healthy) [13,14,15,16], as well as end-to-end systems that offer the advantage of directly mapping the raw speech signal or time–frequency spectrograms to output labels [6,17,18,19,20,21,22]. In this respect, several architectures have been successfully used, such as multilayer perceptrons (MLPs) [13,15], a combination of convolutional neural networks (CNNs) and MLPs [6,17,18], recurrent neural networks (RNNs) [19], a combination of CNNs and long short-term memory (LSTM) networks [16,20], or combinations of time-distributed 2D-CNN and 1D-CNN [21,22]. Furthermore, some of these architectures have been implemented by following a transfer learning (TL) strategy to adapt models from the already-grained storing knowledge on similar problems [18,23,24,25,26].

Most reported DL methods have shown valuable results in the binary categorisation between healthy controls (HCs) and PD when trained with a single dataset. However, even though end-to-end DL approaches have shown promising results in extracting abstract and discriminative features, the available corpora with voice/speech material from PD patients usually contain a small number of speakers (usually less than 100 subjects). This has led researchers focused on applying DL methods to combine data from several sources, which were recorded in different conditions and from speakers with different demographic characteristics (including their mother tongue). To this respect, and in order to address the generalisation capabilities of trained models, the authors in [21,27,28] presented cross-dataset experiments, reporting significant drops in precision of more than 20 absolute points when using different corpora for testing and training. As shown in [29,30], although the combination of multiple datasets is intended to model the representation space better and to avoid overfitting due to data scarcity, it can also induce certain unwanted behaviours, since DL approaches typically make use of shortcut learning strategies capable of reducing the training loss function by learning characteristics associated with the dataset (e.g., the language, microphone, recording equipment, acoustic environment, etc.) but not necessarily related with the specific phenomenon under analysis (i.e., the presence of PD). Despite this fact, which highlights a significant limitation of DL methods, also demonstrating a noticeable degradation in their discriminative capabilities across corpora, the most recent work continues to compare models based solely on their accuracy in a single corpus.

**Figure 1:**
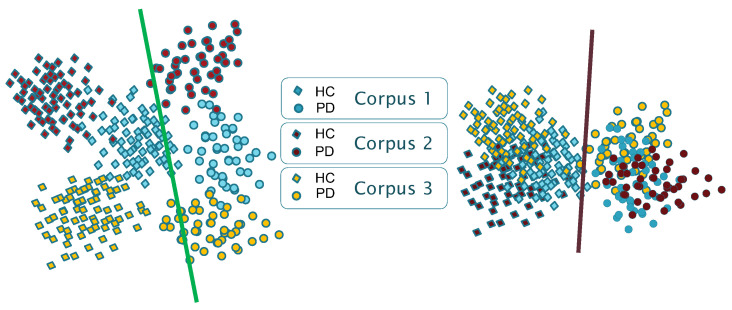
Schematic representation of the clustering effect in a 2D mapping with three corpora (classification into HC/PD with linear decision boundaries). **Left**: corpus-dependent clustering; **Right**: Corpus-independent clustering.

Thus, as reported for other applications [29], the results presented in the state-of-the-art suggest that the available models suffer from certain biases and shortcuts. As schematically shown in Figure 1, models show corpus-dependent clusterings when data from different datasets are combined for training. This is mainly due to the small size of the corpora typically used, but also due to the specific language and channel characteristics of each corpus (differences in the recording equipment, recording parameters, room acoustics, ambient noise, external sounds, etc.). Consequently, a reasonable assumption is that each database encapsulates specific characteristics that could be associated with a distinct domain (including the language), and that DL architectures follow shortcut learning strategies to model the specific conditions of the available corpora. This suggests that trained models capture, in part, distinguishing characteristics between domains (i.e., corpora) rather than only capturing generic features that effectively discriminate the underlying pathology (which is supposed to be corpus and language independent).

As a result, robust model representations are needed to mitigate these undesired factors. We argue that an embedding domain adaptation strategy applied to the learning representation process would narrow the existing gap between different corpora. The goal is to train discriminative and invariant models to domain changes. From now on, we will talk about the domain in reference to a certain corpus.

In view of the aforementioned, this work focuses on studying the potential of domain adversarial (DA) training methods [30,31] to provide more generic and reliable models for the automatic screening of PD using voice and speech. Results are expected to be independent of the specific characteristics of the corpus. Although with a different strategy to identify the domain, the DA training has been explored in other speech applications, such as automatic speech recognition [32], speech emotion recognition [33,34], spoken language identification [35], accent speech recognition [36], and voice conversion [37]. In the context of PD screening, a first attempt of applying domain adaptation was presented in [38], which used speaker identity-invariant representations from a single database (i.e., each domain is assumed to correspond to one speaker), but excluding multi-dataset scenarios.

For this purpose, this paper presents a comparison of three DL architectures widely used in the literature, but trained following DA methods, under the assumption that each corpus corresponds to a different domain. This is carried out using four corpora, which contain speakers with different mother tongue, demographic, and dialectal variations, and who were recorded under different recording conditions.

The main contributions of this work are enumerated next:

- The development of more generalizable methods for the screening of PD by refining the existing end-to-end DL approaches using voice and speech.
- The integration of DA training as a viable strategy to develop models that retain their discriminative capacity to detect PD using the voice/speech from diverse datasets.
- The development of artificial models for the screening of a PD invariant to the mother tongue, demographic, and dialectal variations, as well as corpus recording conditions.
- The reduction in existing learning shortcuts due to the domain (i.e., the corpus) for the screening of PD from voice/speech.
- The reduction in the variability and inter-domain divergence computed within each class (i.e., voice/speech from PD or HC speakers).

The paper is organised as follows. Section 2 introduces the material and methods used in this paper. Section 3 mainly describes the results and an analysis of the different experiments. Section 4 presents a discussion of the results. And Section 5 ends with the conclusions.

## 2. Materials and Methods

This section provides detailed information on the materials and stages involved in the methodology.

The acoustic material used in this work is the sustained phonation of the vowel /a/, and a classic diadochokinetic (DDK) exercise (repetition of the syllable sequence /pa-ta-ka/). This acoustic material is available in the four corpora employed. The differences between the utterances of speakers from the different datasets are supposed to be mainly due to language or dialectal variations.

For modelling, three deep learning (DL) models were chosen based on their promising results presented in previous studies [21,26,39]. For each architecture, a baseline model was developed by selecting the best hyperparameters for a binary categorisation of healthy and pathological voices using a database of voice disorders. The networks trained with this dataset were later adjusted to detect PD, following a TL strategy. For this purpose, the first layer was frozen, and each model was re-trained for the new task. The performance of these three baseline networks was evaluated within each corpus and with combined datasets. Finally, the accuracy of the baseline architectures was compared with their DA counterparts.

### 2.1. Corpora

The four PD databases employed in this work have been widely used in the literature. In the following, they are referred to as PD-GITA [40], PD-Neurovoz [27], PD-Czech [41], and PD-German [42]. Each database includes both PD patients and HC subjects, with patients diagnosed and labelled by neurologists following the Unified Parkinson’s Disease Rating Scale (UPDRS) and the Hoehn and Yahr scale (H&Y). These databases vary in demographics and sizes, as summarised in Table 1. In all cases, the data recordings were conducted under controlled ambient conditions, with the staff instructing each participant to perform various speech tasks, including the sustained phonation of vowel /a/ and a DDK exercise. They have a reasonably good balance of age and sex.

These databases were freely available or transferred for research purposes by the respective authors. A brief description of each database is given below.

**Table 1:** Demographic information, including gender, mean age (standard deviation), and age ranges, for the PD-GITA, PD-Neurovoz, PD-Czech, and PD-German corpora.

Corpus	Subjects				Age (Years)				Age Range (Years)			
	Female		Male		Female		Male		Female		Male	
	PD	HC	PD	HC	PD	HC	PD	HC	PD	HC	PD	HC
PD-GITA	25	25	25	25	60.1 (7.8)	60.7 (7.7)	62.2 (11.2)	61.2 (11.3)	44–75	43–76	33–77	31–86
PD-Neurovoz	21	23	23	24	70.0 (8.6)	69.5 (7.4)	67.0 (10.2)	61.0 (7.5)	56–86	58–86	41–80	53–77
PD-Geman	41	44	47	44	67.2 (9.7)	62.6 (15.2)	66.7 (8.7)	63.8 (12.7)	27–84	28–85	44–82	26–83
PD-Czech	20	20	30	30	60.1 (8.7)	63.5 (11.1)	65.3 (9.6)	60.3 (11.5)	41–72	40–79	43–82	41–77

#### 2.1.1. PD-GITA

A total of one hundred native Colombian Spanish speakers (50 HC and 50 PD) were recruited to create the PD-GITA speech database. They were recorded at Clínica Noel in Medellín, Colombia. The recording protocol included various tasks, such as sustained phonations of the vowels (/a/, /e/, /i/, /o/, and /u/), diadochokinetic evaluation, repetition of different words, both complex and simple sentence repetitions, reading a text, and delivering a monologue. The recordings were sampled at 44.1 kHz with 16-bits of resolution, using a dynamic omnidirectional microphone (Shure, SM 63L). More details about the database are given in [40].

#### 2.1.2. PD-Neurovoz

Ninety-one adult speakers (44 HC and 57 PD), native speakers of Castilian Spanish, were recruited for the speech recordings in this database. The samples were collected by the Otorhinolaryngology and Neurology Departments of Gregorio Marañón Hospital in Madrid, Spain. This corpus includes recordings of sustained vowels, DDK tests, six fixed sentences, and running speech describing a picture. Speech signals were recorded using an AKG C420 headset microphone connected to a phantom power preamplifier. The sampling rate was 44.1 kHz, and the quantisation was carried out with 16-bits. Detailed information about this database can be found in [27,43].

#### 2.1.3. PD-German

A total of one hundred seventy-six German native speakers were recruited to create this database (88 HC and 88 PD). The speech recordings were collected at a hospital in Bochum, Germany. They were engaged in various speech tasks, including sustained vowels, DDK tests, reciting five sentences, reading an 81-word text, and delivering a monologue. The recording samples were obtained using a headset microphone (Plantronics Audio 550 DSP; Plantronics Inc., Santa Cruz, CA, USA), placed 5 cm from the participant’s mouth, with a sampling rate of 44.1 kHz and 16-bits of resolution. More detailed information about the PD-German database can be found in [42].

#### 2.1.4. PD-Czech

A total of one hundred native Czech speakers participated in this study (50 HC and 50 PD). The PD-Czech database was compiled at the General University Hospital in Prague, Czech Republic. The speech tasks included in this corpus consist of the sustained phonation of vowel /a/, DDK tests, reading of a set of 80 distinct Czech words, and delivering a monologue. All samples were recorded using an external condenser microphone positioned approximately 15 cm from the participants’ mouth. The sampling rate was 48 kHz with a 16-bit resolution. For additional information about this corpus, please refer to [41].

#### 2.1.5. Saarbrücken Voice Disorders Database

The Saarbrücken Voice Disorders Database (SVDD) [44] was used to train the initial models that were later adapted using a TL strategy. This database was compiled by personnel from the Institute of Phonetics at the University of Saarland in Germany. The corpus contains voice recordings from 687 healthy controls and 1355 individuals with different pathological conditions. All of them are native German speakers. The SVDD includes recordings of the sustained vowels /a/, /i/, and /u/ under four different loudness conditions (normal, high, low, and low–high–low), vowels with rising–falling pitch, and one sentence. All samples were recorded at 50 kHz with 16-bits of resolution. The SVDD database is available online at [44].

### 2.2. Data Pre-Processing

**Figure 2:**
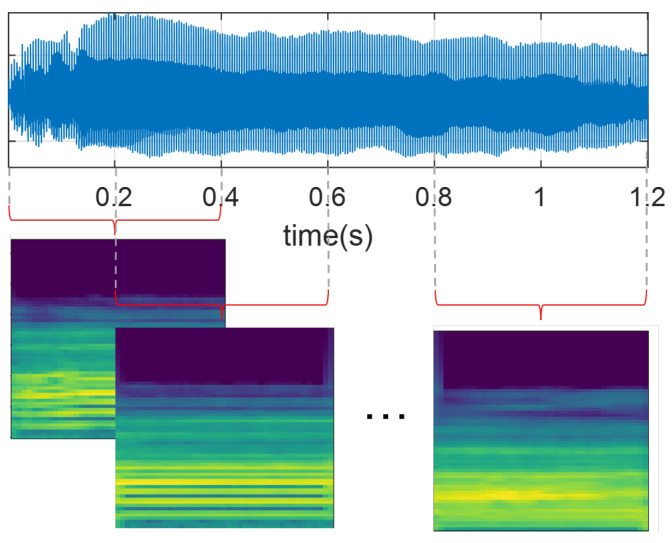
Graphical representation of the audio segmentation procedure and computation of the Mel spectrograms.

All audio recordings from the PD-Czech and SVDD databases were resampled at 44.1 kHz to ensure a consistent sampling rate concerning PD-GITA and PD-Neurovoz. Signals were then segmented into 400 ms segments with a 50% of overlap, as illustrated in Figure 2. The window size was selected to preserve all subject recordings, considering that some recordings of vowel /a/ in the PD-GITA dataset have a maximum duration of 490 ms. Additionally, all speech signals were normalised based on the maximum absolute amplitude values.

Subsequently, all recordings were transformed into the time–frequency domain using Mel-scale spectrograms [20,21,38]. For this study, we computed the Mel-scale representations using windows that were 40 ms long for the sustained phonation of vowel /a/ to preserve the quasi-stationarity assumption and ensure independence from the location of pitch pulses within the segment, as discussed in [45]. For DDK tests, fifteen-millisecond-long frames were extracted, which yielded optimal results in a previous study [46]. In both cases, the hop length was set to 10 ms, and the number of Mel bands was set to 65. Finally, the resulting Mel-scale spectrogram images (with their amplitude in dBs), each sized 65 × 41 pixels, were normalised following a z-score scaling.

### 2.3. Domain Adversarial Networks

**Figure 3:**
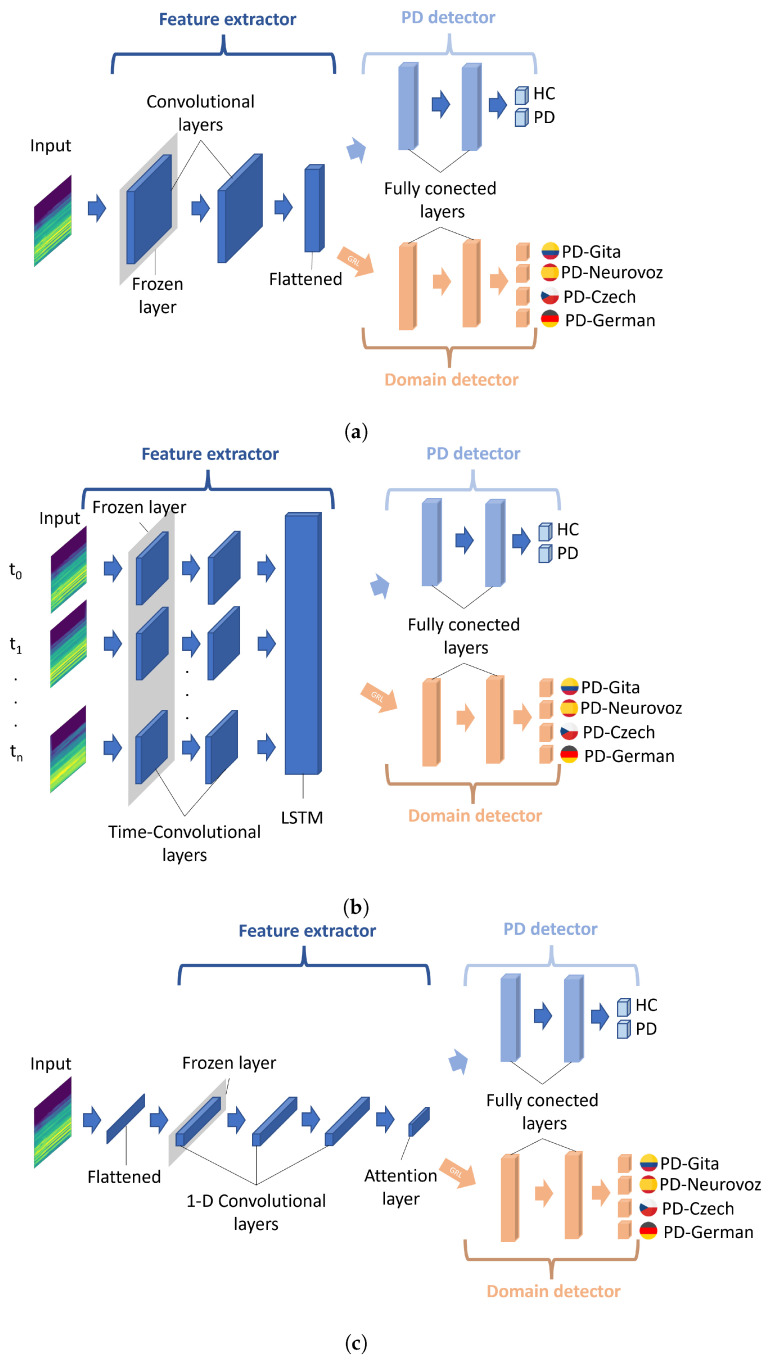
Diagram of the DL architectures adapted for DA training. (**a**) 2D-CNN; (**b**) Time-CNN-LSTM; (**c**) 1D-CNN. A gradient reversal layer (GRL) is included between the feature extractor and the domain detector. Figures adapted from [31].

Figure 3 illustrates the three DL architectures adapted for DA training: 2D-CNN, Time-CNN-LSTM, and 1D-CNN. The choice of these base architectures is strongly motivated by their success in classifying PD from voice and speech, as demonstrated in [6,16,17,20,21,26]. Additionally, their requirements in terms of memory and graphics processing unit (GPU) are reasonable, and they are well documented and well understood. The choice of these architectures is also motivated by a potential comparison of the results with previous works in the state-of-the-art.

Each architecture was adapted, following the DA neural network framework proposed in [31]. Consequently, all architectures consist of three modules: a feature extractor, a PD detector, and a domain detector (i.e., a corpus detector). The feature generator serves as a shared network between both the PD and the domain detectors, receiving the Mel spectrograms as input. The PD detector’s role is to discriminate the primary learning task (i.e., binary classification between PD and HC), trying to minimise the classification error. However, the domain detector aims to maximise the error due to the dataset to which the observation belongs, i.e., it promotes the information extracted from the spectrograms to be unable to discriminate among corpora. The domain detector is linked to the feature extractor through a gradient reversal layer (GRL), which maintains the input’s integrity during forward propagation and reverses the gradient by multiplying it with a negative scalar during backpropagation, as outlined in [30]. The application of the gradient reversal ensures that the feature distributions across the four domains (datasets) become more similar, which would lead to the generation of domain-invariant features.

In order to obtain more generalisable results, a TL strategy was also followed from other models created using large corpora trained for similar classification tasks. To this respect, the baseline architecture (consisting of both the feature extraction module and the PD detection module) underwent a pretraining to classify healthy speakers and patients with voice disorders. This is carried out using the vowels available in the SVDD database. Despite the fact that this dataset contains voices associated with various pathologies, previous research has shown that a similar strategy can significantly improve the accuracy of PD detection [18,23,24,25,26]. TL was implemented herein through freezing only the initial layer of the feature extractor (highlighted in grey in Figure 3). The freezing of more layers was refrained to ensure an adequate number of parameters that could be used for the fine-tuning stage during the DA training.

In the three networks, the architecture of the PD detector and the domain detector is similar, consisting of two fully connected layers with a dropout layer in between to regularise the weights. ReLu activation was used in the first hidden layers, and a softmax activation function was applied for classification. Regarding the feature extractor module, this component varies among the architectures. A brief description of them and their specific configurations is presented below.

#### 2.3.1. 2D-CNN Network

CNN is a well-known architecture for classifying data presented as a multi-dimensional array, such as greyscale and colour images, time–frequency representations of audio, and videos [47]. Therefore, CNNs have been widely employed for the screening of PD, converting the audio signals into time–frequency representations [6,17,18,21,26,39]. The CNN architecture implemented in this work is illustrated in Figure 3a. This network comprises two-dimensional convolutional layers, where each convolutional layer is followed by a batch normalisation, a ReLU activation function, max pooling (filter size: 3 × 3), and a dropout layer. Subsequently, the dynamic features obtained by the first module are flattened to connect with the first fully connected layer of both the PD detector and the domain detector. Since similar architectures have been used in the literature but with varying filter sizes, in this work, they are set using the cross-validation strategy discussed in Section 2.4. The 2D-CNN network architecture takes one Mel-scale spectrogram as input at a time. To determine the ultimate patient classification, a post-processing stage calculates the joint probability for all spectrograms obtained from a single patient and assigns the patient to the class with the highest joint probability.

#### 2.3.2. Time-CNN-LSTM Network

The combination of time-distributed CNN and LSTM networks enables the mapping of time-varying features from a multi-dimensional source [48]. In this architecture, the input is treated as a temporal sequence, where a time-distributed convolution layer applies the same transformation to each input frame. The role of the LSTM is to extract global temporal features. In this study, the architectural configuration is illustrated in Figure 3b. Unlike the 2D-CNN network architecture, in this case, the input is a sequence of *n* consecutive frames of Mel spectrograms from the same recording, with zero padding applied when the signal lengths are insufficient to complete *n* frames; a masking strategy removes the zeros during the processing phase. Hence, this network comprehensively analyses all patient information in a single forward pass, eliminating the necessity for any post-processing to arrive at a final prediction for the patient. The initial stage comprises two time-distributed 2D-CNN layers. Similarly to the previous network, each convolutional layer is followed by batch normalisation, a ReLU activation function, max pooling (with a filter size of 3 × 3), and a dropout layer. Subsequently, the flattened outputs of the time-distributed CNN serve as the input sequential features for a bidirectional LSTM. The hidden states of the LSTM cells are used as input features for both the PD and the domain detector. Similar to the previous architecture, the size of the convolutional filters is set during the experimental phase.

#### 2.3.3. 1D-CNN Network

This architecture was proposed in [49]. It consists of several 1D-CNN layers, and its temporal output is summarised through a convolutional attention mechanism, as illustrated in the scheme in Figure 3c. The first layer of the network corresponds to a flatten operator aiming to fit together the 2D spectrogram inputs with the 1D convolutional layers. The network comprises three convolutional blocks with kernel sizes of 5, 11, and 21, respectively, with max pooling layers (kernel size of 6) in between. Each convolutional block includes a one-dimensional convolution, followed by batch normalisation, a ReLU activation function, and a dropout layer. In the last 1D-CNN layer, half of the filters are subjected to a time-wise softmax activation, which functions as an attention mechanism for the other half of the filters [49]. Subsequently, the attention output serves as input features for both the PD and the domain detector. Similar to the 2D-CNN network, a post-processing stage to obtain the ultimate prediction per patient is also used.

### 2.4. The Experimental Setup

For all experiments, the training and evaluation were performed following a stratified speaker-independent 10-fold cross-validation strategy, ensuring that there was no overlap of speakers across different folds. First, the hyperparameters of the baseline architectures were tuned using Talos [50] with 10-folds extracted from the SVDD dataset. Table 2 summarises the hyperparameter search space. The model with the best performance on the validation set among the 10 folds was selected for all experiments, including for the DA training, where the domain detector network parameters were set to the same values as the PD detection network parameters. All models were evaluated in terms of accuracy, sensitivity, specificity, and F1 score.

The different architectures were trained using the Stochastic Gradient Descent (SGD) algorithm with cross-entropy as the loss function. When training with imbalanced datasets, such as the SVDD corpus and for the domain detector, a weighted cross-entropy loss function was used, where the weights were automatically set to compensate for the data imbalance. A learning rate schedule was used, initialised as 0.1.

The models were trained using a workstation equipped with two NVIDIA GeForce RTX3090 GPUs with 24 gigabytes of VRAM memory each.

## 3. Results

The first set of experiments was carried out to compare the performance of the baseline architectures with and without TL for each dataset. The first objective was to replicate the accuracies reported in previous work and to demonstrate the boosting effect of TL enhancing the PD detection, particularly for the sustained vowel /a/.

For the second series of experiments, the four speech corpora were combined to train and test both the baseline architectures and their DA versions. The features extracted by the models were categorised by class (PD and HC) and domain (PD-GITA, PD-Neurovoz, PD-German, and PD-Czech) and plotted on a two-dimensional map using the Stochastic Neighbour Embeddings distributed by the t (t-SNE) tool [51]. This visualisation tool helps to evaluate the clustering effect of the extracted features and lets us compare the results of the baseline and DA architectures. To quantify the differences in the distribution of features labelled between domains within each class, a metric was also calculated using the Kullback–Leibler (KL) divergence between the intra-class domain feature distributions [52], as well as another metric based on the trace of the covariance matrix (TCM) of the intra-class features [53].

### 3.1. Baseline Results

Table 3 presents a comparison of the results obtained for the three baseline architectures and for the four available corpora. TL, in most cases, shows improvements in accuracy, F1 score, sensitivity, and specificity, which is consistent with previous research in the field [18,23,24,26]. Also, the accuracy obtained aligns with that obtained in previous studies. For example, in [26], using a CNN adapted with TL, the mean accuracy scores (with standard deviation) for the classification of PD reached 72 (17.5), 83.7 (13.5), and 71.0 (22.3) for PD-GITA, PD-German, and PD-Czech DDK recordings, respectively. In this work, the highest accuracy for the same datasets was 80.1 (7.6), 64.0 (10.4), and 74.8 (10.2), respectively. Additionally, in [16], the reported precision for Time-CNN-LSTM and 1D-CNN using vowel /a/ from the PD-GITA corpus was 76.2 and 72.0, respectively, while in this work, it was 72.4 and 72.6, respectively (for comparison purposes, note the large standard deviations).

In general, as shown in Table 3, the DDK tests exhibit better performance compared to the sustained vowel /a/. The accuracy obtained for the DDK tests in some datasets reached values above 80%, while, for vowel /a/, the maximum accuracy remains below 75%. A similar trend was also observed in [46]. Furthermore, it is important to consider the significant variability of the results across different datasets and tasks for the three baseline networks. For example, in the case of the sustained vowel /a/ using the PD-Neurovoz dataset, the highest performance was achieved with a 2D-CNN. However, when evaluating DDK utterances within the same dataset, the Time-CNN-LSTM outperformed the others. On the contrary, the 1D-CNN yielded the highest accuracy for DDK tests when applied to the PD-Czech dataset. These results highlight the limitations of developing PD detection models based solely on a single corpus or a single task.

### 3.2. Domain Adversarial Results

**Figure 4:**
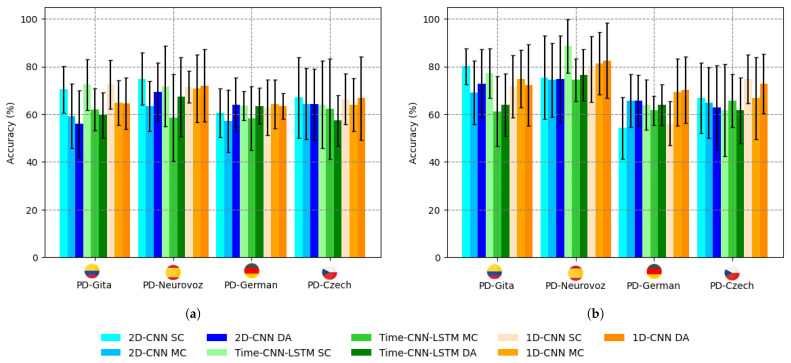
Bar chart of PD detection accuracy for the baseline networks using a single corpus (SC), mixed corpora (MC), and DA, for: (**a**) q sustained vowel /a/; (**b**) DDK tests.

The results for accuracy, F1-score, sensitivity, and specificity obtained for both the baseline architectures and their corresponding DA versions are presented in Table 4. In this series of experiments, the architectures were trained and tested by combining data from all four datasets. The precision of these experiments is also summarised in Figure 4 compared to those obtained for the baseline architectures using a single corpus, as reported in Table 3 using TL. The bar graphs in Figure 4 reveal a noticeable trend: a decrease in accuracy for the baseline architectures trained and tested, mixing the different corpora available, which is also consistent with the results reported in previous studies [28,54]. However, the DA network effectively mitigates this trend. This effect is particularly noticeable in the case of the 1D-CNN with DA training, where the accuracy with mixed corpora either exceeds or closely matches that obtained with a single dataset (except for vowel /a/ from PD-GITA).

**Figure 5:**
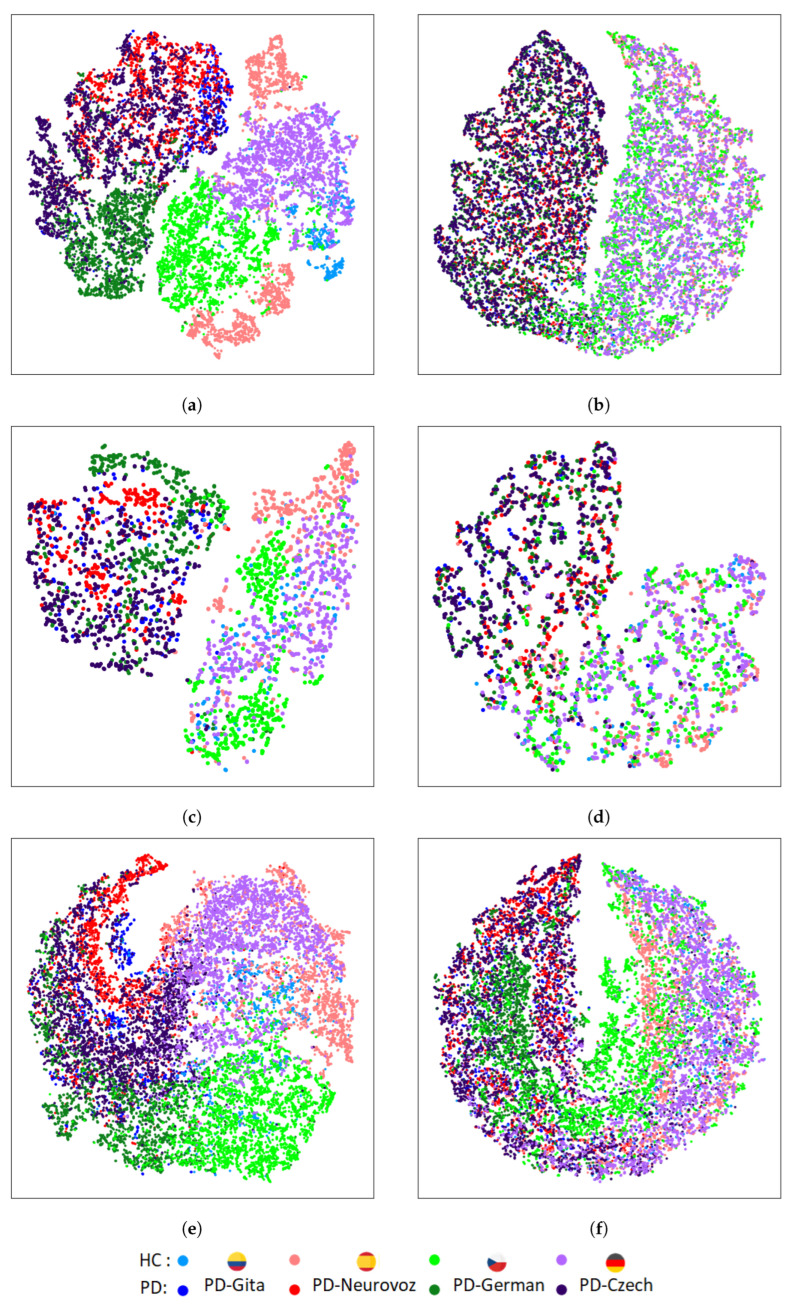
T-SNE of the extracted features in the last layer of the PD detector module for the training set using DDK tests: (**a**) 2D-CNN; (**b**) DA 2D-CNN; (**c**) Time-CNN-LSTM; (**d**) DA Time-CNN-LSTM; (**e**) 1D-CNN; (**f**) DA 1D-CNN.

**Figure 6:**
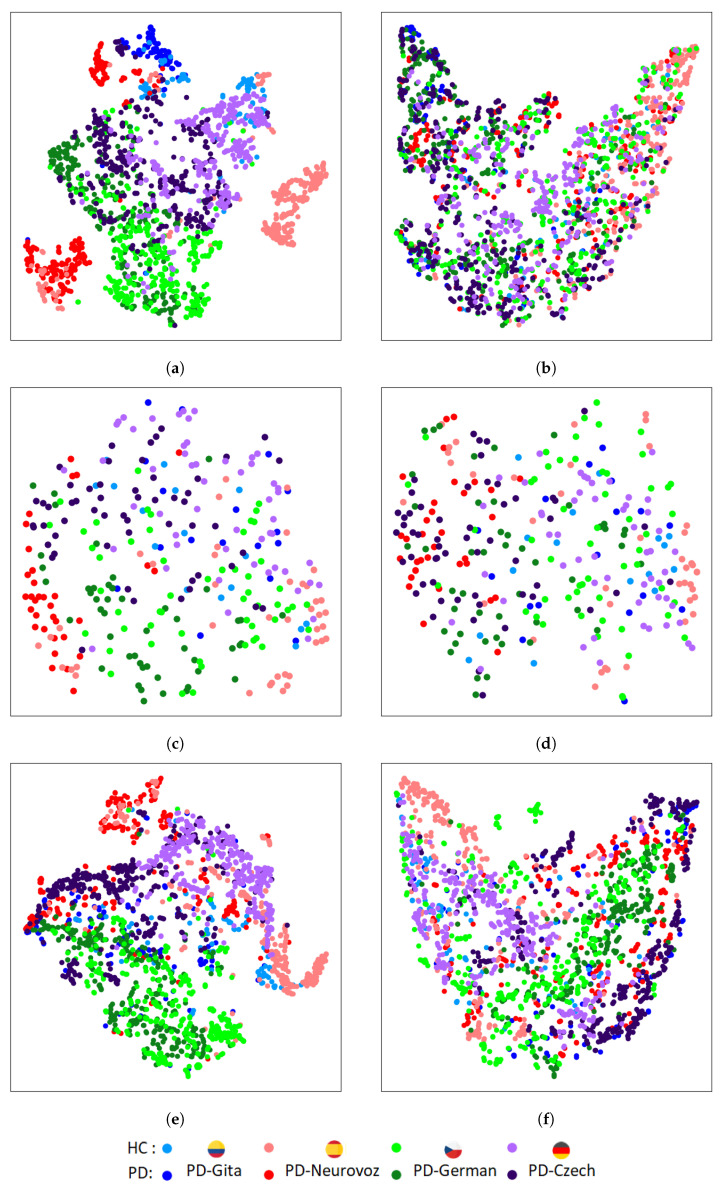
T-SNE of extracted features in the last layer of the PD detector module for the validation set trained using DDK tests: (**a**) 2D-CNN; (**b**) DA 2D-CNN; (**c**) Time-CNN-LSTM; (**d**) DA Time-CNN-LSTM; (**e**) 1D-CNN; (**f**) DA 1D-CNN.

Figure 5 and Figure 6 depict the t-SNE representations of the features extracted by the baseline and DA networks for the training and validation sets, respectively, using DDK tests. For each architecture, the t-SNE plot corresponds to the fold that reported the best accuracy during the validation process. Figure 5 represents the t-SNE mapping of the features extracted during training for the three baseline architectures. The mapping evidences distinct clusters associated with each corpus. On the other hand, Figure 6, represents the t-SNE mappings for the DA networks, showing only two clusters: one for HC, and another for PD. The same trend is observed for the validation set (Figure 6), especially for the 2D-CNN and 1D-CNN. A similar behaviour was observed when using the sustained vowel /a/. However, in this case, the clusters in the validation set were less evident due to the lower discrimination capability and consequently reduced classification performance obtained.

In contrast to the results for the baseline architectures, t-SNE mappings report that DA networks are able to extract features with similar distributions across the four domains, identifying only two main clusters associated with both classes (i.e., HC and PD), and removing undesirable corpus-dependent clusterings. These similarities were also quantified using the KL divergence between the intra-class domain feature distributions, and the TCM for the intra-class domain features. The results of the KL divergence are presented in Table 5, where DA networks exhibit lower distances between pairs of domain distributions compared to the baseline architectures. Table 6 presents the results using the TCM for the intra-class domain features, showing that the baseline architectures present higher values than the DA networks, indicating a greater spread for each class.

## 4. Discussion

DL methods have shown promise in extracting discriminative features for the detection of PD from voice and speech. However, due to ethical constraints and the large amount of resources needed to collect the required corpora, existing studies often suffer from limited training data, leading researchers to combine recordings from different sources. However, this mixing of datasets results in a loss of precision when networks are trained with one dataset and tested on another. This is attributed to certain biases and shortcuts learnt by DL networks related to dataset-specific characteristics, which include not only the language of the speakers, but also the channel characteristics of each corpus (i.e., recording equipment, recording parameters, room acoustics, ambient noise, external sounds, etc.).

On the other hand, previous analyses of end-to-end DL architectures for PD classification using voice and speech recordings have mainly relied on comparing architectures trained with a single corpus. However, the experiments conducted on individual and multiple datasets reveal that the models obtained lack generalisability: although they demonstrate high performance when trained on a single database, their performance drops significantly when trained using multiple corpora.

A detailed analysis of the results provided in the state-of-the-art shows that traditional end-to-end DL architectures are not able to learn the expected corpus-independent characteristics of PD. This is due to the presence of shortcuts in the learning process, which are manifested as corpus-dependent clusterings of the features extracted. As aforementioned, they are mainly explained not only due to the small size of the corpora used, but also due to language differences, demographics of the population, and variances in the channel characteristics of each corpus.

In order to address these issues, this work explores the use of DA training as a strategy to develop new artificial models capable of detecting PD from voice and speech by combining data from different corpora. In this regard, this paper uses three off-the-shelf DL architectures and their DA counterparts, the latter developed under the assumption that each corpus corresponds to a different domain. The architectures developed were evaluated with sustained vowels and DDK recordings extracted from four different corpora with a variety of dialects or languages, demographics, and channel characteristics. Additionally, in order to obtain more robust models, this work combines DA training with a TL approach.

The results reported based on the objective distance metrics used in this article (KL divergence between the intra-class domain feature distributions, and TCM for the intra-class domain features) and based on the t-SNE plots of the features extracted from the baseline architectures trained using several datasets evidence the aforementioned extremes. They show a clear clustering related to the variability of the domain (i.e., the corpus), rather than a clustering based only on discriminatory characteristics of PD. It is important to note that this limitation is more evident for 2D-CNN, which has been widely used for the detection of PD [6,23,24,25,26].

On the other hand, results reported based on the objective distance metrics used and based on the t-SNE plots extracted from the DA networks show that learnt features from the four corpora follow a similar clustering for both classes considered (i.e., HC and PD). Such clustering has demonstrated to be corpus independent and does not rely on language differences and potential variances in the channel characteristics.

These results align with our preliminary work in which we implemented a DA CNN trained with DDK recordings extracted from PD-GITA and PD-Neurovoz [54]. The new experiments presented in this paper, including new corpora, different tasks, and new architectures, provide new evidence that DA training improves the generalisability of the models obtained.

On a different note, the study also indicates that architectures trained using sustained vowels are less efficient compared to those trained with DDK tests. These findings are consistent with the research in [46], which emphasises the importance of articulation for the automatic detection of PD. The absence of articulatory information from sustained vowels explains why the accuracy is below 70% (Figure 4a) for standard training or 75% using a TL approach (Table 3).

The results suggest that DA training introduces a certain degree of interpretability in the artificial models, but this technique still relies on DL architectures with limited explainability. On the other hand, despite the encouraging results obtained, the corpora used are still too small to ensure that the results could be extrapolated to larger datasets.

## 5. Conclusions

We discussed some challenges in developing accurate PD screening models using voice and speech data, particularly when combining training data from different sources. In this regard, we suggest DA training as a potential solution to mitigate shortcut learning effects and dataset-specific biases, as well as to improve model generalisation across different corpora. This makes the models developed more interpretable, thus improving the possibility of transfer to clinical practice.

This study investigates three end-to-end DL approaches, along with their respective DA networks, for the detection of PD in a multi-corpus scenario. Our analysis of the extracted features revealed that traditional DL methods perform corpus-dependent clusterings of the features, hindering the generalisation capabilities of DL models for PD detection. On the other hand, the study provides evidence suggesting that DA strategies mitigate this effect.

In light of these findings, we consider DA to be an effective approach for creating robust corpus-independent PD detection models from voice and speech. Our exploration has highlighted the potential of DA methods as a promising approach to accomplish this objective. Thus, they provide a practical pathway toward creating language-independent and corpus-invariant PD detection models.

This research contributes to ongoing efforts to improve the detection of PD and paves the way for further investigations into domain adaptation techniques in medical speech analysis. We believe that the insights gained from this study will be valuable for the advancement of the field.

## Figures and Tables

**Table 2:** Search space for the hyperparameters used to train the baseline architectures.

Hyperparameter	2D-CNN	Time-CNN-LSTM	1D-CNN	Values
Training Batch size	✓	✓	✓	16, 32, 64
Dropout rate	✓	✓	✓	0.2, 0.5
Depth of conv. layer	✓	✓	✓	32, 64, 128
Units of each fully connected layer	✓	✓	✓	16, 32, 64
Kernel size of conv. layer I	✓	✓		4, 6, 8
Kernel size of conv. layer II	✓	✓		5, 7, 9
Number of units LSTM layer		✓		16, 32, 64
Number of frames (*n*)		✓		3, 5, 7, 9

**Table 3:** Classification results following a 10-fold cross-validation. The mean (standard deviation) of the accuracy (Acc.), F1-score, sensitivity (Sen.), and specificity (Spe.) were calculated for the three baseline architectures, both with and without TL. These metrics were evaluated separately for each corpus (PD-GITA, PD-Neurovoz, PD-German, and PD-Czech) and for two tasks: vowel /a/ and DDK tests.

			Vowel /a/				DDK Tests			
Corpus	Network	TL	Acc.	F1-Score	Sen.	Spe.	Acc.	F1-Score	Sen.	Spe.
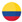 PD-GITA	2D-CNN	✓	68.6 (15.4)	69.9 (14.1)	70.0	70.3	78.8 (13.4)	79.0 (13.4)	81.8	76
			70.5 (9.8) *	68.7 (11.6)	68.3	74.0	**80.1 (7.6)**	80.8 (6.7)	84.7	76.0
	Time-CNN-LSTM	✓	66.7 (11.5)	68.4 (9.7)	68.0	67.3	75.5 (13.5)	74.9 (18.3)	72.9	76.3
			**72.4 (10.6)**	71.9 (12.5)	70.0	76.1	**77.3 (10.4)**	78.2 (12.0)	76.8	76.3
	1D-CNN	✓	70.0 (11.0)	71.1 (10.4)	76.3	64.7	64.0 (11.8)	72.7 (7.4)	94.0	34.0
			**72.6 (10.2)**	73.4 (9.3)	77.0	68.0	**71.7 (13.1)**	77.0 (9.2)	92.0	52.0
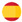 PD-Neurovoz	2D-CNN	✓	73.9 (12.0)	67.9 (24.5)	68.5	79.0	**77.8 (15.5)**	76.8 (19.8)	80.5	76.0
			**74.9 (10.9)**	71.1 (14.7)	68.5	81.0	75.4 (17.5)	71.8 (27.3)	73.5	78.5
	Time-CNN-LSTM	✓	62.8 (16.7)	61.5 (17.3)	62.5	62.5	83.5 (13.3)	86.5 (10.0)	85.5	81.7
			**71.8 (17.0)**	69.6 (21.1)	68.9	74.3	**88.6 (11.2)**	89.5 (10.7)	88.7	88.3
	1D-CNN	✓	71.3 (10.5)	68.6 (11.5)	66.0	77.0	77.8 (16.6)	73.4 (27.0)	73.0	83.0
			**71.5 (6.6)**	70.4 (8.9)	73.5	70.5	**79.0 (13.8)**	76.7 (18.5)	75.5	83.0
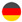 PD-German	2D-CNN	✓	58.9 (10.1)	59.5 (13.8)	64.2	52.8	54.2 (10.2)	53.3 (13.8)	54.4	54.2
			**60.6 (10.3)**	61.6 (11.9)	65.4	55.3	**54.3 (12.9)**	53.0 (17.4)	55.1	53.2
	Time-CNN-LSTM	✓	60.3 (10.0)	60.3 (10.1)	62.0	60.1	59.3 (13.1)	59.7 (16.8)	66.8	52.7
			**63.6 (6.1)**	64.6 (6.0)	67.5	60.8	**64.0 (10.4)**	64.1 (12.4)	70.7	58.1
	1D-CNN	✓	59.4 (7.6)	60.8 (9.2)	64.4	54.2	52.7 (7.8)	47.5 (10.1)	44.1	61.0
			**62.9 (11.7)**	62.7 (10.8)	62.4	63.3	**56.1 (9.2)**	55.7 (9.4)	55.8	56.3
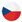 PD-Czech	2D-CNN	✓	66.0 (17.0)	60.6 (19.5)	58.0	73.5	64.9 (15.5)	61.7 (15.2)	58.0	71.5
			**67.0 (17.0)**	60.0 (21.5)	57.5	75.5	**66.9 (14.7)**	61.5 (18.1)	56.0	77.5
	Time-CNN-LSTM	✓	62.4 (20.3)	50.9 (28.6)	50.8	75.6	58.5 (15.4)	55.6 (18.1)	56.7	65.8
			**64.1 (18.3)**	57.5 (20.4)	56.1	73.3	**61.7 (19.4)**	60.5 (20.9)	61.2	67.3
	1D-CNN	✓	64.5 (11.2)	57.3 (15.2)	57.0	71.5	71.9 (15.9)	69.5 (19.3)	66.0	78.0
			**66.4 (10.7)**	57.9 (23.6)	61.0	71.5	**74.8 (10.2)**	73.0 (11.9)	70.0	79.5

* Best results in boldface.

**Table 4:** Classification results following a 10-fold cross-validation. Mean percentages (with standard deviation) of the accuracy (Acc.), F1-score, sensitivity (Sen.), and specificity (Spe.) for both baseline and DA networks trained with vowel /a/ and DDK tests, and using mixed datasets from PD-GITA, PD-Neurovoz, PD-German, and PD-Czech.

			Vowel /a/				DDK Tests			
Network	DA	Test	Acc.	F1-Score	Sen.	Spe.	Acc.	F1-Score	Sen.	Spe.
2D-CNN		PD-GITA	59.3 (13.6) **	59.9 (10.6)	60.7	61.3	69.2 (13.4)	67.3 (18.5)	68.8	70.0
		PD-Neurovoz	63.5 (10.5)	59.7 (16.1)	62.0	67.0	74.4 (15.5)	73.7 (18.8)	75.5	73.0
		PD-German	57.2 (13.0)	57.5 (14.1)	59.5	54.2	65.8 (11.1)	65.1 (11.9)	65.1	65.8
		PD-Czech	**64.3 (14.9)**	52.6 (20.6)	48.5	77.5	**64.8 (14.8)**	56.8 (25.1)	52.0	78.0
	✓	PD-GITA	56.0 (14.1)	55.5 (16.5)	58.0	59.0	**72.9 (14.4)**	71.8 (16.2)	72.0	74.0
		PD-Neurovoz	**69.4 (12.3)**	67.4 (11.9)	65.5	73.5	**74.9 (18.2)**	72.9 (20.3)	72.0	78.5
		PD-German	**64.1 (11.2)**	63.3 (12.0)	62.4	65.6	**65.8 (10.7)**	62.6 (12.1)	58.6	72.9
		PD-Czech	64.2 (14.9)	54.7 (25.6)	55.5	71.5	62.8 (17.7)	55.2 (26.5)	52.0	74.0
Time-CNN-LSTM		PD-GITA	**62.0 (8.9)**	62.4 (14.3)	67.7	60.7	61.2 (14.6)	57.7 (22.7)	60.3	62.0
		PD-Neurovoz	58.6 (18.3)	58.3 (24.3)	65.5	52.5	74.4 (9.0)	73.3 (10.1)	71.0	78.0
		PD-German	58.4 (13.4)	57.8 (12.0)	56.7	59.6	61.7 (6.1)	60.5 (7.0)	60.6	62.2
		PD-Czech	**62.3 (21.1)**	50.7 (31.6)	48.0	75.0	**65.7 (11.1)**	62.0 (13.2)	58.0	73.5
	✓	PD-GITA	59.6 (9.4)	53.6 (16.1)	53.0	72.0	**64.1 (13.1)**	59.4 (20.1)	60.7	68.0
		PD-Neurovoz	**67.3 (16.6)**	67.0 (18.7)	73.0	62.5	**76.5 (10.8)**	77.3 (10.9)	79.5	74.0
		PD-German	**63.5 (7.5)**	62.3 (10.9)	63.2	63.1	**64.0 (8.5)**	63.1 (9.0)	62.9	64.6
		PD-Czech	57.4 (10.6)	47.0 (20.9)	46.0	67.5	61.7 (13.8)	58.3 (17.3)	56.0	67.5
1D-CNN		PD-GITA	**64.9 (9.4)**	65.1 (10.4)	68.7	63.0	**74.9 (12.1)**	74.7 (13.6)	77.0	74.0
		PD-Neurovoz	70.8 (14.2)	71.8 (13.2)	77.0	66.0	81.4 (13.0)	82.7 (11.9)	86.5	75.5
		PD-German	**64.2 (10.2)**	64.0 (10.8)	64.6	63.1	69.3 (14.1)	69.7 (13.8)	72.2	65.8
		PD-Czech	64.1 (11.1)	57.2 (15.4)	56.5	71.5	66.8 (17.2)	62.4 (27.1)	64.0	69.5
	✓	PD-GITA	64.5 (10.8)	64.3 (15.4)	70.0	60.3	72.2 (17.1)	69.7 (19.7)	67.0	78.0
		PD-Neurovoz	**72.0 (15.2)**	69.4 (20.7)	73.0	72.0	**82.6 (15.7)**	82.7 (15.8)	82.5	83.0
		PD-German	63.4 (5.45)	63.9 (8.3)	67.0	60.0	**70.2 (14.0)**	72.8 (11.8)	78.1	62.0
		PD-Czech	**66.8 (17.5)**	59.4 (25.7)	59.5	73.5	**72.8 (12.6)**	67.9 (20.2)	66.0	79.5

** Best results in boldface.

**Table 5:** KL divergence between intra-class feature distributions for domain pairs: PD-GITA (G), PD-Neurovoz (N), PD-German (Ger), and PD-Czech (C). These values represent the mean for 10 folds.

			Vowel /a/						DDK Tests					
Network	DA	Label	G-N	G-Ger	G-C	N-Ger	N-C	Ger-C	G-N	G-Ger	G-C	N-Ger	N-C	Ger-C
2D-CNN		HC	43.6	35.4	41.9	42.8	41.6	43.4	28.3	24.7	19.6	30.7	29.5	20.1
		PD	42.3	37.8	43.3	43.4	39.8	43.4	26.3	24.1	20.5	27.4	25.0	20.1
	✓	HC	28.3	26.0	27.3	30.0	30.3	28.1	8.9	9.5	7.6	9.7	9.6	8.1
		PD	28.0	25.6	30.1	28.4	29.5	30.4	10.2	8.0	8.5	9.0	8.2	7.6
Time-CNN-LSTM		HC	27.9	23.5	27.9	28.5	24.0	28.8	12.9	7.4	4.7	14.3	14.0	8.4
		PD	24.1	23.6	27.1	27.9	25.1	30.1	11.3	10.9	6.4	13.2	11.6	9.2
	✓	HC	18.9	19.5	20.3	21.4	21.1	21.0	6.4	3.3	3.7	6.7	7.1	3.6
		PD	17.6	19.8	21.4	20.6	21.7	22.8	8.9	5.7	4.2	6.3	5.6	5.6
1D-CNN		HC	27.5	23.5	25.5	30.6	26.4	27.9	19.5	19.0	19.3	23.9	18.7	23.0
		PD	27.5	23.4	29.5	28.1	28.6	29.2	17.1	15.8	15.0	19.9	16.2	17.8
	✓	HC	22.5	22.8	21.9	24.5	23.9	22.6	12.0	13.2	11.9	14.7	11.7	14.7
		PD	21.3	18.7	22.4	22.3	23.3	24.5	10.6	11.1	11.5	11.8	11.9	12.9

**Table 6:** Mean TCM values for the intra-class domain features calculated for 10 folds.

		Vowel /a/		DDK Tests	
Network	DA	HC	PD	HC	PD
2D-CNN	✓	43.6	42.3	50.0	35.4
		11.3	13.9	10.2	10.4
Time-CNN-LSTM	✓	4.2	3.7	3.2	3.0
		3.4	3.1	2.6	2.4
1D-CNN	✓	2.2	2.8	2.9	2.5
		1.4	1.5	1.4	1.4

## Data Availability

The models used in this work were trained using the PD-GITA [40]. PD-Neurovoz [27], PD-German [42] and PD-Czech [41]. Data are available from the authors upon request. The SVDD corpus is available online [44]. The source code is available at https://github.com/BYO-UPM/DA_PD (accessed on 8 October 2023).

## Abbreviations

The following abbreviations are used in this manuscript:

1D-CNN	One-Dimensional Convolutional Neural Network
2D-CNN	Two-Dimensional Convolutional Neural Network
C	PD-Czech
CNN	Convolutional Neural Network
DA	Domain Adversarial
DL	Deep Learning
DDK	Diadochokinetic test
G	PD-GITA
Ger	PD-German
GRL	Gradient Reversal Layer
HC	Healthy control
H&Y	Hoehn and Yahr Scale
KL	Kullback–Leibler
LSTM	Long Short-Term Memory
MC	Mixed Corpora
MLP	Multilayer Perceptron
N	PD-Neurovoz
PD	Parkinson’s Disease
RNN	Recurrent Neural Network
SC	Single Corpus
SGD	Stochastic Gradient Descent
SVDD	Saarbrücken Voice Disorders Database
TCM	Trace of the Covariance Matrix
Time-CNN-LSTM	Time-Distributed CNN and LSTM Networks
TL	Transfer Learning
t-SNE	Stochastic Neighbour Embeddings Distributed by t
UPDRS	Unified Parkinson’s Disease Rating Scale
